# Humic Substances Contribute to Plant Iron Nutrition Acting as Chelators and Biostimulants

**DOI:** 10.3389/fpls.2019.00675

**Published:** 2019-05-22

**Authors:** Laura Zanin, Nicola Tomasi, Stefano Cesco, Zeno Varanini, Roberto Pinton

**Affiliations:** ^1^Dipartimento di Scienze AgroAlimentari, Ambientali e Animali, Università degli Studi di Udine, Udine, Italy; ^2^Faculty of Science and Technology, Free University of Bozen-Bolzano, Bolzano, Italy; ^3^Dipartimento di Biotecnologie, Università di Verona, Verona, Italy

**Keywords:** Fe complex, Fe chelates, fulvic acids, root uptake, strategy I, strategy II, water-extractable humic substances (WEHS)

## Abstract

Improvement of plant iron nutrition as a consequence of metal complexation by humic substances (HS) extracted from different sources has been widely reported. The presence of humified fractions of the organic matter in soil sediments and solutions would contribute, depending on the solubility and the molecular size of HS, to build up a reservoir of Fe available for plants which exude metal ligands and to provide Fe-HS complexes directly usable by plant Fe uptake mechanisms. It has also been shown that HS can promote the physiological mechanisms involved in Fe acquisition acting at the transcriptional and post-transcriptional level. Furthermore, the distribution and allocation of Fe within the plant could be modified when plants were supplied with water soluble Fe-HS complexes as compared with other natural or synthetic chelates. These effects are in line with previous observations showing that treatments with HS were able to induce changes in root morphology and modulate plant membrane activities related to nutrient acquisition, pathways of primary and secondary metabolism, hormonal and reactive oxygen balance. The multifaceted action of HS indicates that soluble Fe-HS complexes, either naturally present in the soil or exogenously supplied to the plants, can promote Fe acquisition in a complex way by providing a readily available iron form in the rhizosphere and by directly affecting plant physiology. Furthermore, the possibility to use Fe-HS of different sources, size and solubility may be considered as an environmental-friendly tool for Fe fertilization of crops.

## Introduction

Soil HS are generally considered as the result of the partial degradation and re-synthesis of organic material, especially of plant residues. They originate from polymerization/polycondensation of phenolic compounds, mainly deriving from microbial lignin degradation. As a consequence, soil HS have a strong aromatic nature; nonetheless, during the condensation process a number of organic molecules including aliphatic chains, peptides, amino acids, fatty acids and sugars can be incorporated, thus forming substances from medium to high molecular weight (Stevenson, 1994). Soil HS could also originate from associations of relatively small humic molecules linked together by hydrophobic interactions and hydrogen bonds (Piccolo, 2002). Humic molecules of different molecular masses can bind together forming a supramolecular humic network; the degree of aggregation may depend on the pH, ionic strength and mineral composition of the solution (Garcia-Mina, 2007; Esfahani et al., 2015).

These processes imply that HS of different molecular size and solubility are present in the soil. Some fractions are present in the soil solution, thus being able to directly interact with plant roots (Chen and Schnitzer, 1978; Gerke, 1997). These latter soluble HS are considered as part of the DOM (Bolan et al., 2011).

Humic substances are routinely extracted from the soil with alkaline solutions and then can be operationally fractionated, based on their different water solubility, into humic (HA) and fluvic (FA) acids (Stevenson, 1994).

Due to their heterogeneity, the molecular structure of soil HS cannot be unequivocally identified. Nevertheless, it has been clearly defined that the presence of some functional groups within their structure are responsible for the observed indirect and direct effects on plant growth and nutrition (Nebbioso and Piccolo, 2011; Muscolo et al., 2013; García et al., 2016a). Indirect effects refer to changes in the chemical and physical properties of soil and rhizosphere, while direct ones indicate actions on plasma membrane (PM)-bound activities and plant metabolic pathways (Varanini and Pinton, 2001; Nardi et al., 2002; Zandonadi et al., 2013; Canellas and Olivares, 2014; Rose et al., 2014; Olaetxea et al., 2018).

The occurrence of HS in soils, as representative of natural organic matter evolution, has been questioned; rather it has been proposed that they are the result of the alkali-based extraction procedure (Lehmann and Kleber, 2015). While this aspect is still under debate (Gerke, 2018; Olk et al., 2019), it is noteworthy that humic-like molecules have been extracted from soils treated with mild extractants (Hayes, 2006), found in aquatic environments (Alberts and Takács, 2004), peat water extracts and soil leachates (Pinton et al., 1997; Vujinovic et al., 2013).

Despite being the chemical nature of HS still controversial, it has been unequivocally demonstrated that organic materials of different origin can provide available Fe to plants as a results of Fe complexation by humic molecules (Chen et al., 2004a; Bocanegra et al., 2006; Kovács et al., 2013; Cieschi et al., 2017). Furthermore, soluble Fe-HS complexes could be formed and directly used by the plants (Pandeya et al., 1998; Pinton et al., 1999). The capacity of HS to complex metals and affect the mechanisms of nutrient acquisition and plant metabolism provide evidence for a multifaceted role of these organic fractions on Fe nutrition.

In the present work, we will summarize recent reports on the role of HS in plant Fe nutrition that can be attributed to their chelating and biostimulant effect, with a special emphasis on effects exerted by the water-soluble fractions.

## Effects of Humic Substances on Iron Availability

Humic substances are able to form stable complexes with metal micronutrients, due to the presence in their structure of oxygen-, nitrogen- and sulfur-containing functional groups. This, in turn, would help maintaining micronutrients in solution and/or in bioavailable forms at pH values found in most soils (Senesi, 1992; Tipping, 2002). In the case of Fe, highly stable HS complexes mainly involve O-containing groups (carboxylic and phenolic groups) (Senesi, 1992; Tipping, 2002). More recently it was shown that carboxylic acids in aliphatic domains are also involved in Fe(III)-HS complexation (Fuentes et al., 2013).

The stability order of the complexes formed between metals and humic acids has been determined through potentiometric titration and follows the Irving-Williams series. Evaluation of stability constants for metal-HS complexes (Garcia-Mina et al., 2004) showed values somewhat lower than those observed for complexes between Fe and synthetic chelating agents (e.g., EDTA, EDDHA; Lucena, 2003) or organic compounds of biological origin (e.g., organic acids, siderophores, PS, phenols) (von Wirén et al., 2000; Crowley, 2001; Ryan et al., 2001; Mimmo et al., 2014).

Stability and solubility of the complexes are both affected by pH and molar ratio between micronutrients and HS (Chen et al., 2004a; Garcia-Mina, 2006). A high stability would be favored in the 5–9 pH range by a low metal:HS ratio, while a high solubility would be favored by alkaline pH and a low metal:HS ratio. This implies that plants growing in calcareous soils with limited Fe availability could benefit from the formation of stable and soluble Fe-HS complexes (Cieschi and Lucena, 2018), as well as of insoluble complexes with high molecular weight HS (Colombo et al., 2014).

Humic substances can affect Fe availability also through the stabilization of amorphous Fe oxides by high molecular weight humic fractions (Schwertmann, 1991). The poorly crystalline Fe phases, co-precipitated with insoluble HS (IHS) and maintained for a long period in this form, can represent a reservoir of iron suitable, *via* ligand mobilization, for plant Fe nutrition (Colombo et al., 2012, 2014).

The ability of HS to complex Fe can also be important for phosphorous nutrition, since phosphate can be bound to HS by Fe bridges (Gerke, 2010; Urrutia et al., 2013). This process would increase phosphate availability; in fact, complexation of Fe by ligands released by plant roots could promote uptake of both nutrients (Gerke, 1993; Urrutia et al., 2014).

Humic substances are known to be redox reactive and capable of chemically reducing metals including Fe^3+^ (Skogerboe and Wilson, 1981; Struyk and Sposito, 2001). Reduction of Fe^3+^ occurs at significant levels at pH values lower than 4; at higher pH values reduction is limited by formation of complexes between Fe^3+^ and humic molecules. It has been shown that dissolved and solid-phase HS can accelerate Fe(III)-oxide reduction in sediments (Nevin and Lovley, 2002; Roden et al., 2010) and bioreduction of Fe(III) minerals in soils (Rakshit et al., 2009), by shuttling electrons from bacteria to oxide surfaces.

## Role of Humic Substances as Natural Chelates

Besides delaying the Fe crystallization processes, HS can contribute to Fe nutrition via formation of water-soluble Fe-HS complexes, which can move in the soil and reach the roots (Pandeya et al., 1998; Garcia-Mina et al., 2004; Chen et al., 2004b). These complexes would act as natural Fe-chelates interacting with plant uptake mechanisms. Using a water-extractable humic fraction (WEHS), purified from a water extract of sphagnum peat, it was demonstrated that a Fe-WEHS complex could be obtained by interaction between the humic fraction and a poorly soluble Fe form (Cesco et al., 2000). Fe-WEHS complex could, in turn, be used by Fe-deficient Strategy-I and Strategy-II plants. Uptake by Strategy-I plants could occur via the Fe(III) reduction-based mechanism (Pinton et al., 1999), while in Strategy-II plants, a ligand exchange between WEHS and PS was conceivably involved (Cesco et al., 2002). Uptake of ^59^Fe from ^59^Fe-WEHS complex was measured even at pH values compatible with those found in calcareous soils (Cesco et al., 2002; Tomasi et al., 2013) and the same held true for root Fe(III) reduction in Strategy-I plants (Tomasi et al., 2013; Zamboni et al., 2016). The recovery of Fe-deficient plants following the treatment with Fe-WEHS was paralleled by a stimulation of the acidification capacity of roots, a component of the Fe-deficiency response in Strategy-I plants (Pinton et al., 1999; Tomasi et al., 2013).

Iron from ^59^Fe-WEHS complex appeared to be accumulated in higher amount within the plant as compared with other natural chelates, such as ^59^Fe-citrate or ^59^Fe-PS (Tomasi et al., 2013; Zamboni et al., 2016). Furthermore, a higher translocation of Fe to the leaves was observed in Fe-deficient Strategy-I plants supplied with ^59^Fe-WEHS (Tomasi et al., 2009; Zanin et al., 2015) as compared with the other two natural Fe-chelates. This behavior was accompanied by an increase of Fe content in the xylem sap (Tomasi et al., 2009). In ^59^Fe-WEHS-treated cucumber plants Fe was more rapidly allocated into the leaf veins and transferred to interveinal cells (Zanin et al., 2015). Similar effects were reported by Bocanegra et al. (2006) who observed a rapid translocation of Fe from roots to leaves of plants treated with a low molecular weight humic fraction. These results indicate that HS could affect Fe nutrition not only by increasing the metal availability in the soil and in the rhizosphere, but also acting on the mechanisms involved in its uptake and its translocation within the plant.

Supply of HS or Fe-HS complexes has also been shown to affect expression of genes related to Fe-uptake mechanisms. Providing a Fe-WEHS complex to Fe-deficient tomato plants induced an up-regulation of root Fe(III)-chelate reductase (*LeFRO1*) and Fe transporter genes, *LeIRT1* and *LeIRT2* (Tomasi et al., 2013). The increase in transcript abundance was faster and reached a higher level than when Fe-citrate or Fe-PS were used. Aguirre et al. (2009) showed that the treatment of cucumber plants with HS purified from leonardite induced a transient up-regulation of genes involved in the Strategy-I uptake mechanism, that is *CsHA2*, *CsFRO1* and *CsIRT1*, in cucumber roots. These effects were associated with an increase of the root Fe(III) chelate-reductase activity. Billard et al. (2013) showed that a humic fraction isolated from black peat could induce the up-regulation of the *IRT1* gene in both the roots and leaves of rapeseed plants. These results were correlated to a significant increase of the Fe concentration in leaves.

Interestingly, also genes involved in Fe uptake in leaves (*CsFRO1*, *CsIRT1*, *CsNRAMP*) were up-regulated following Fe-WEHS supply to Fe-deficient cucumber plants, as compared with Fe-PS-fed plants (Zanin et al., 2015). The localization of *CsFRO1*, *CsIRT1* transcripts was evident next to the midveins, while *CsNRAMP* expression was detected in the overall mesophyll region, supporting a role of this later gene in the Fe distribution within the whole leaf tissue.

Genome-wide transcriptional analysis revealed that the early response to Fe supply of Fe-deficient tomato plants was strongly influenced by the nature of the chelating agent (Zamboni et al., 2016). In fact, Fe-citrate and Fe-PS modulated, respectively the expression of 728 and 408 genes, showing a fast down-regulation of molecular mechanisms induced by Fe deficiency. On the other hand, Fe-WEHS did not determine relevant changes in the root transcriptome with respect to the Fe-deficient plants, suggesting that roots did not sense the restored cellular Fe accumulation. This behavior would account for the higher Fe accumulation in Fe-WEHS treated plants.

## Effects of Humic Substances on Root Growth and Functions

**FIGURE 1:**
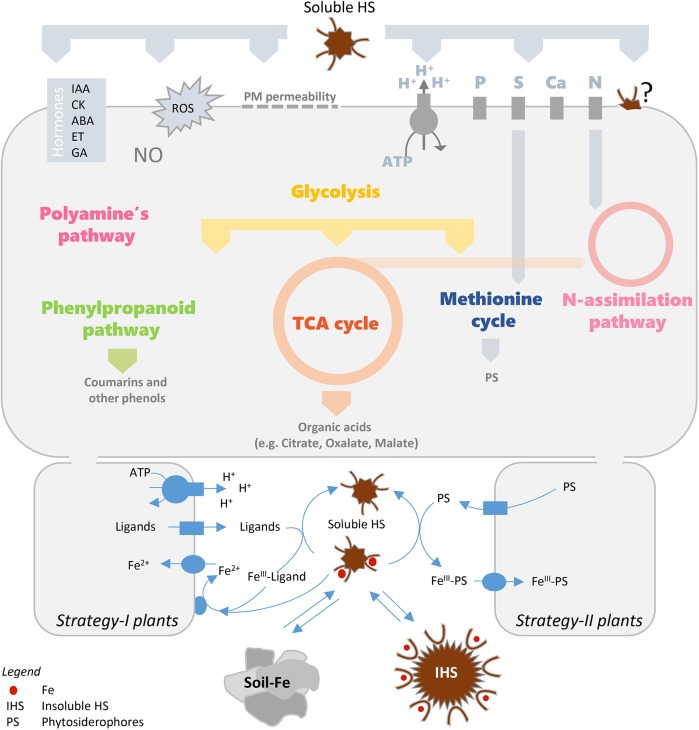
Physiological and molecular plant responses induced by HS (in the upper part) and role of Fe-HS complexes on Fe plant nutrition (in the lower part). Schematic drawings of Strategy-I and Strategy-II components on PM of root cells are shown (for Strategy I: a proton pump, a ligand efflux transporter, an Fe transporter and an Fe^III^-chelate reductase are depicted in the left; for Strategy II: a ligand efflux transporter and an Fe-chelate transporter are depicted in the right). ABA, abscisic acid; CK, cytokinin; ET, ethylene; GA, gibberellic acid; IAA, indole-3-acetic acids; IHS, insoluble HS; NO, nitric oxide; PS, phytosiderophores; ROS, reactive oxygen species; TCA, tricarboxylic acid; PM, plasma membrane; GS/GOGAT, glutamine synthase/glutamine oxoglutarate aminotransferase).

Treatments of plants with HS have been shown to induce changes in root morphology and modulate plant membrane activities related to nutrient acquisition, pathways of primary and secondary metabolism, hormonal and reactive oxygen balance (Varanini and Pinton, 2001; Nardi et al., 2002; Canellas and Olivares, 2014; Olaetxea et al., 2018; Figure 1). These effects, which vary depending on the origin, molecular size and chemical characteristics of HS, suggest an action of these organic fractions on growth promotion and stress resistance in plants.

Many authors observed that plants treated with HS of different origin were able to induce proliferation of lateral roots and root hairs (Canellas et al., 2002; Nardi et al., 2002). This behavior has been related to the activation of signaling pathways involving phytohormones, especially auxin, nitric oxide, Ca^2+^ and ROS (Trevisan et al., 2010; Zandonadi et al., 2010; Mora et al., 2012; Ramos et al., 2015; García et al., 2016b,c). Up-regulation of auxin-regulated genes (Trevisan et al., 2011) and modulation of genes coding for enzymes involved in hormone metabolisms (Zanin et al., 2018) suggest that HS might influence the steady-state equilibrium of different plant hormones. However, stimulation of root growth was observed also independently of hormonal changes (Schmidt et al., 2007; Mora et al., 2012), suggesting that other signals might be involved in the morphological modifications elicited by HS.

A recognized target of HS action is the root PM H^+^-ATPase (Zandonadi et al., 2016). Evidence for activation of the PM proton pump has been observed both at transcriptional and post-transcriptional level and related to proton extrusion (Varanini et al., 1993; Canellas et al., 2002) and uptake of ions, such as nitrate (Pinton et al., 1999; Quaggiotti et al., 2004; Tavares et al., 2017), phosphate (Jindo et al., 2016) and sulfate (Jannin et al., 2012). Besides ion uptake, HS have been shown to promote nitrogen assimilation (Mora et al., 2010; Jannin et al., 2012; Vaccaro et al., 2015; Zanin et al., 2018), carbon metabolism (glycolysis and Krebs cycle; Nardi et al., 2007; Trevisan et al., 2011) and synthesis of secondary metabolites, such as phenylpropanoids (Schiavon et al., 2010; Jannin et al., 2012; García et al., 2016c).

In addition to the stimulation of proton release, HS have been shown to affect rhizodeposition. Humic acids promoted release of anionic species close to region of root acidification [apolar sugars from maize roots (Puglisi et al., 2009), impacting soil microbial community in the rhizosphere (Puglisi et al., 2013)]. Increase in root growth was accompanied by a greater release of low molecular weight exudates from maize plants treated with HS (Canellas et al., 2019). On the other hand, it has been reported that organic acids, such as those released by the roots, could disaggregate supramolecular structure of HS releasing low molecular weight humic fractions (Piccolo et al., 2003), which in turn might exert their effects on roots. Regarding this point is noteworthy that accumulation of HS at the root surface and in the apoplast has been observed (García et al., 2012; Kulikova et al., 2014). Furthermore, HS fractions obtained from rhizospheric soil showed different chemical characteristics to those isolated from bulk soil (D’Orazio and Senesi, 2009).

Effects of HS on root growth (signaling pathways), ion uptake (primary and secondary membrane transporters), primary metabolism (nitrogen and carbon), secondary metabolism (phenylpropanoids) and root exudation might be important for Fe acquisition and could improve the response of plants to Fe deprivation (Figure 1).

## Conclusion and Perspective

Plenty of papers in the last decades have proven the capability of HS, isolated from different organic sources, to affect plant growth, nutrition and metabolism.

In natural soils, these substances, due to their heterogeneity and polydispersity, can be present as co-precipitates with mineral parts (e.g., Fe-oxides and clays) or in the solution where they contribute a considerable portion of the DOM.

Low-molecular-weight and water-soluble fractions have been shown to affect functionality of ion transporters operating on the PM of root cells, acting both at transcriptional and post-transcriptional level. This evidence has been achieved mostly using controlled experimental conditions, such as isolated HS and hydroponically grown plants. Conceivably, these HS could directly interact with plant roots, microorganisms and soil particles in the rhizosphere. Thus, study of structural and chemical characteristics of HS present in soil solution and in the rhizosphere are needed to allow the transfer of knowledge obtained in controlled systems to real soil/rhizosphere conditions. This would help to shed light on the direct contribution of HS to plant nutrition and growth and on their usefulness in the field. Evidence of a relationship between chemical structural characteristics of HS obtained from different sources and having variable molecular complexity and the biological effects they exert on plants has been already provided. This kind of studies can now be performed using new analytical techniques thus allowing a full characterization of HS based on their origin, either natural or anthropogenic.

Concerning Fe nutrition, these aspects would be very useful considering the dual role that has been attributed to HS, as chelating compounds and biostimulants (Table 1). The capability of HS to form stable complexes with Fe and to directly affect Fe-acquisition mechanisms would account for the relative contribution of Fe-HS complexes to plant Fe nutrition as compared to other Fe-complexes naturally occurring in the rhizosphere.

**Table 1:** Reports focusing on the role of humic substances in iron plant nutrition.

Humic substances		Crop		HS Treatment	Objectives	Actions	References
	
Source	Fraction/ Size	Species	Organ				
Humate (from leaf compost)	N/A	*Solanum lycopersicum*	Shoots, Roots	supply of humates (100 mg L^-1^ dm^3^)	Influence of sodium humate on the uptake or some ions by tomato seedlings	Facilitated the Fe transport from roots to shoots and stimulated the root uptake of K^+^, Rb^+^, Mg^2+^ and PO_4_^3-^, while strongly inhibited the Cl^-^ uptake	Gumiński et al., 1983
FA (from sphagnum peat)	WEHS	*Cucumis sativus, Hordeum vulgaris*	Plants	supply of ^59^Fe-WEHS (1 μM Fe; 5 mg C_org_ L^-1^ WEHS) up to 3 days	Strategy-I and Strategy-II plant capabilities to use Fe complexed by WEHS	cucumber plants (Strategy I) utilize Fe-WEHS, presumably via reduction of Fe(III)-WEHS by PM Fe reductases, while barley plants (Strategy II) use an indirect mechanism involving ligand exchange between WEHS and PS	Cesco et al., 2002
HA (from mollisol)	N/A	*Helianthus annuus, Hordeum vulgare*	Plants	^59^Fe-HA complex and EDTA or DTPA (0.1 mM) for 1, 4, or 14 days	Study the release and diffusion of Fe from Fe-HA chelates and its availability to growing plants	EDTA and DTPA attracted and chelated substantial amounts of the ^59^Fe bonded by the HA, presumably by a ligand exchange process	Bocanegra et al., 2004
HA (from mollisol)	HA_100,000_ (>100 KDa); HA_10,000_ (<10 kDa)	*Helianthus annuus*	Plants	supply of ^59^Fe-HA (50–100 mg L^-1^) for 15 days	Plant uptake of iron chelated by humic acids of different size	Rapid translocation of Fe to the leaves; the small size HA10,000 and EDTA were the most efficient in affecting transport of Fe from root to leaf tissue	Bocanegra et al., 2006
HA (from leonardite)		*Cucumis sativus*	Roots	supply of HA (2, 5, 100, and 250 mg C_org_ L^-1^ up to 92 h; 40 μM of Fe were added as Fe-EDTA	Dose effect of HA on Fe-deficient response in cucumber plants	HA treatments transiently up-regulated in roots *CsFRO1*, *CsIRT1* and *CsHA2* expression and increased the Fe(III) chelate-reductase and PM H+-ATPase activity	Aguirre et al., 2009
FA (from sphagnum peat)	WEHS	*Solanum lycopersicum*	Leaves	supply of Fe-WEHS (1 μM Fe; 5 mg C_org_ L^-1^ WEHS) up to 24 h	study on mechanisms induced by Fe-WEHS at the leaf level	efficient use of Fe complexed by WEHS, at least in part, also the activation of Fe-acquisition mechanisms operating at the leaf level (upregulation of *LeFRO1*, *LeIRT1* and *Ferritin2* genes)	Tomasi et al., 2009
Insoluble HS (from Leonardite) and FA (from sphagnum peat)	HMW and WEHS	*Cucumis sativus*	Plants	supply of Fe-HS (0.1–10 μM Fe; 5 mg C_org_ L^-1^ HS) up to 11 days	efficiency of Fe-IHS complexes in alleviating Fe chlorosis	use of Fe insoluble high-molecular weight complexes (Fe-IHS) as an effective product to correct the Fe nutritional disorder	Colombo et al., 2012
high molecular weight HS (HA7 extract from black peat)	0.96–68 kDa	*Brassica napus*	Leaves, Roots	supply of HA7 (100 mg C_org_ L^-1^ HA7) up to 1, 3 or 30 days	Effect of HA treatment on rapeseed nutrition	HA7 incresed the Fe content in shoots and induced the expressionof genes coding for *BnIRT1, BnCOPT2, BnNRAMP3*	Billard et al., 2013
water soluble HS (from Leonardite)	WSHS	*Cucumis sativus*	Plants	supply of Fe-WSHS (20 μM Fe; Fe:LN = 1:1.1) for 1 day	study the use of Fe^3+^/Fe^2+^ species in Fe-LN for plant nutrition	Fe^2+^-WSHS use efficiently by plants under hydroponic conditions, while Fe^3+^-WSHS is used more effectively under calcareous soil conditions	Kovács et al., 2013
FA (from sphagnum peat)	WEHS	*Solanum lycopersicum*	Roots	supply of Fe-WEHS (1 μM Fe; 5 mg C_org_ L^-1^ WEHS) up to 24 h	Physiology and molecular response of Fe-deficient plants	increased the ^59^Fe hydroxide solubilization, the ^59^Fe root uptake and gene expression of *LeFRO1* and *LeIRT1* and *LeIRT2*	Tomasi et al., 2013
FA (from sphagnum peat)	WEHS	*Cucumis sativus*	Leaves, Roots	supply of Fe-WEHS (1 μM Fe; 5 mg C_org_ L^-1^ WEHS) up to 5 days	Nutrient allocation in leaves of Fe-deficient lants	Increased root uptake of nitrate, CO_2_ assimilation while changed the allocation of several nutrients from the vascular system (K, Cu, and Zn) or trichomes (Ca and Mn) to the entire leaf blade.	Tomasi et al., 2014
FA (from sphagnum peat)	WEHS	*Cucumis sativus*	Leaves	supply of Fe-WEHS (1 μM Fe; 5 mg C_org_ L^-1^ WEHS) up to 5 days	Iron allocation in leaves of Fe-deficient plants	stimulated the Fe accumulation and allocation in leaves, the upregulation of three transcripts: *CsFRO*, *CsIRT* (both localized next to the midveins) and *CsNRAMP* (in the interveinal area)	Zanin et al., 2015
HA (from leonardite)	N/A	*Triticum aestivum*	Shoots, Roots	Fe–HA (Fe 38.2 mg L^-1^; 98 mg L^-1^ HA)	The effect of Fe-HA on photosynthesis and lipid profile in Fe-deficient plants	Enhanced input of Zn and lipid content in Fe-deficient plants, effect of HAs on the antioxidant status of plants and the plant lipid metabolism	Abros’kin et al., 2016
FA (from sphagnum peat)	WEHS	*Solanum lycopersicum*	Roots	supply of Fe-WEHS (1 μM Fe; 5 mg C_org_ L^-1^ WEHS) for 1 h	Early transcriptomic response in Fe deficient roots	Upregulation of Strategy I components, the feedback regulation of these components does not occured.	Zamboni et al., 2016
humic fraction (from leonardite)	HA, FA	*Glycine max*	Plants	supply of Fe-HS (10–100 μmol Fe pot^-1^) up to 60 days	Study the Fe-HS use efficiency in soybean roots for Fe nutrition under calcareous conditions	Show the effect of HS accumulation on soybean roots in the iron transport from root to shoot and the Fe-biomineralization to form jarosite on the soybean root surface	Cieschi and Lucena, 2018

It is noteworthy that HS induce a “nutrient acquisition response” even when plants are adequately supplied or during the recovery from a deficiency status, affecting functionality and regulation of nutrient uptake mechanisms. The signaling network at the basis of this behavior starts to be elucidated. Furthermore, it has been suggested that the cross-interaction between root exudates and HS might be part of the cross-talk between plant and soil. These features would favor a prompt adaptation of plants to a specific environment.

Another point of interest studying the behavior of HS is their possible use to develop environmentally friendly fertilization tools, being crucial in terms of circular economy. Although their chemical structure is not yet fully understood and the direct transfer of results obtained in controlled conditions to real soil has been questioned, it is quite clear that HS isolated from different organic sources, when added to nutrient solution or to the soil can favor plant nutrition, and especially nitrogen and Fe accumulation. This implies that humic fractions with different chemical and biological properties could be used to tailor HS-based fertilizers with high use efficiency. This tool could be particularly relevant for precision agriculture aimed at limiting external inputs and optimizing the use of natural resources by crops.

## Author Contributions

LZ, NT, and RP wrote the manuscript and SC and ZV critically revised the manuscript. All authors approved the final version of the manuscript.

## Conflict of Interest Statement

The authors declare that the research was conducted in the absence of any commercial or financial relationships that could be construed as a potential conflict of interest. The reviewer YP declared a shared affiliation, though no other collaboration, with one of the authors SC to the handling Editor.

## Abbreviations

ABA: abscisic acid
CK: cytokinins
DOM: dissolved organic matter
ET: ethylene
FA: fluvic acids
FRO: ferric chelate reductase
GA: gibberellic acid
HA: humic acids
HS: humic substances
IAA: indole-3-acetic acids
IHS: insoluble HS
IRT: iron transporter
NO: nitric oxide
NRAMP: natural resistance-associated macrophage proteins
PS: phytosiderophores
ROS: reactive oxygen species
TCA: tricarboxylic acid
WEHS: water extractable humic substances
